# Spleen cells from young but not old immunized mice eradicate large established cancers

**DOI:** 10.1158/1078-0432.CCR-12-0127

**Published:** 2012-03-13

**Authors:** Karin Schreiber, Ainhoa Arina, Boris Engels, Michael T. Spiotto, John Sidney, Alessandro Sette, Theodore Karrison, Ralph R. Weichselbaum, Donald A. Rowley, Hans Schreiber

**Affiliations:** 1 Department of Pathology, The University of Chicago, Chicago, IL 60637; 2 Department of Radiation & Cellular Oncology, The University of Chicago, Chicago, IL 60637; 3 Division of Vaccine Discovery, La Jolla Institute for Allergy and Immunology, La Jolla, CA 92037, USA; 4 Department of Health Studies, The University of Chicago, Chicago, IL 60637; 5 Comprehensive Cancer Center, The University of Chicago, Chicago, IL 60637; 6 Ludwig Center for Metastasis Research, The University of Chicago, Chicago, IL 60637

**Keywords:** Adoptive T cell therapy, tumor eradication, young, old, memory T cells

## Abstract

**Purpose:**

Solid tumors that have grown two weeks or longer in mice and have diameters larger than 1 cm are histologically indistinguishable from autochthonous human cancers. When experimental tumors reach this clinically relevant size, they are usually refractory to most immunotherapies but may be destroyed by adoptive T cell transfer. However, TCR-transgenic T cells and/or tumor cells overexpressing antigens are frequently used in these experiments. Here we studied the requirements for destroying clinical size, unmanipulated 8101 tumors by adoptive cell therapy.

**Experimental Design:**

8101 arose in an old mouse after chronic exposure to UV light. A cancer line was established, which was never serially transplanted. The immunodominant CD8^+^ T cell-recognized antigen of this tumor is caused by a somatic tumor-specific mutation in the RNA helicase p68. 8101 tumors were treated with spleen cells from young naïve, or young and old immunized mice to ascertain the characteristics of immune cells that lead to rejection.

**Results:**

Here we show that the mutant p68 peptide has an exceptionally high affinity to the presenting MHC class I molecule K^b^ and that spleen cells from immunized young syngeneic mice adoptively transferred to Rag^-/-^ or cancer-suppressed euthymic mice eradicate 8101 tumors larger than 1 cm in average diameter and established for several weeks. Spleen cells from naïve young mice or from old and boosted (re-immunized) mice were ineffective.

**Conclusions:**

Relapse-free destruction of large and long-established tumors expressing a genuine very high-affinity tumor-specific antigen can be achieved by using adoptive transfer of lymphocytes from immunized young individuals.

## Introduction

Most human tumors have reached at least 1 cm in diameter and contain at least 10^9^ cancer cells at time of diagnosis. These tumors have been present in the patient for probably many months, if not years (1). However, most experimental tumors used for preclinical studies on the efficacy of immunotherapy do not reach that size, and most strategies fail at later stages of cancer progression (2). Adoptive T cell therapy stands out as the most effective approach and thus offers the highest promise in a clinically realistic setting (2). In several experimental models, mice receiving syngeneic activated T cells rejected established tumors expressing potent, immunodominant artificial antigens (3-5). We have therefore focused on experimental adoptive cell therapy that could be effective against tumors at least 1 cm in diameter and containing ∼10^9^ cancer cells that express natural tumor-specific antigens arisen at an old age.

Most spontaneous human and animal tumors develop in older individuals (6-9). As people age, they are predisposed to developing cancer likely due to increasingly more mutations in their somatic cells as well as a decrease in their ability to mount effective immune responses against these malignant cells. Furthermore, several studies have shown that aged mice fail to reject similar tumor inocula that are eradicated by younger mice (10-12). These distinct age-related immune responses may be due to differences in co-stimulation, regulatory T cells and/or the generation of effector T cells. As a consequence, tumors in elderly patients are likely to harbor tumor-specific antigens, which are not targeted because of deficits of the immune response.

In recent clinical trials, exogenously stimulated, autologous T cells caused regression of large tumor burdens in a fraction of patients (13-17). However, experimental models of adoptive T cell therapy often reject tumors much more effectively than when similar methods are applied to patients. One primary difference between these two observations lies in the age of the donor T cells. The experimental models use T cells often from adolescent or younger donor mice. By contrast, these clinical trials have isolated anti-tumor lymphocytes from the cancer patient. Since cancer patients are usually at a more advanced age, these donor T cells may not function as well as donor T cells from younger individuals. Furthermore, it is not clear to what extent the presence of a tumor could affect the quality of the T cells obtained from cancer patients.

Here we show that adoptive transfer of spleen cells from young but not old immunized mice can eradicate large solid 8101 cancers that have grown for several weeks. These cancer cells express a natural immunodominant target peptide that binds to the presenting MHC class I molecule with nanomolar affinity. These findings suggest that clinically relevant size cancers can be eradicated by adoptive cell therapy also in a more realistic cancer model, and that the age of the immunized lymphocyte donor is critical.

## Results

### Young naïve mice, but not an old naïve mouse, reject a challenge with 8101 tumor fragments

**Figure 1:**
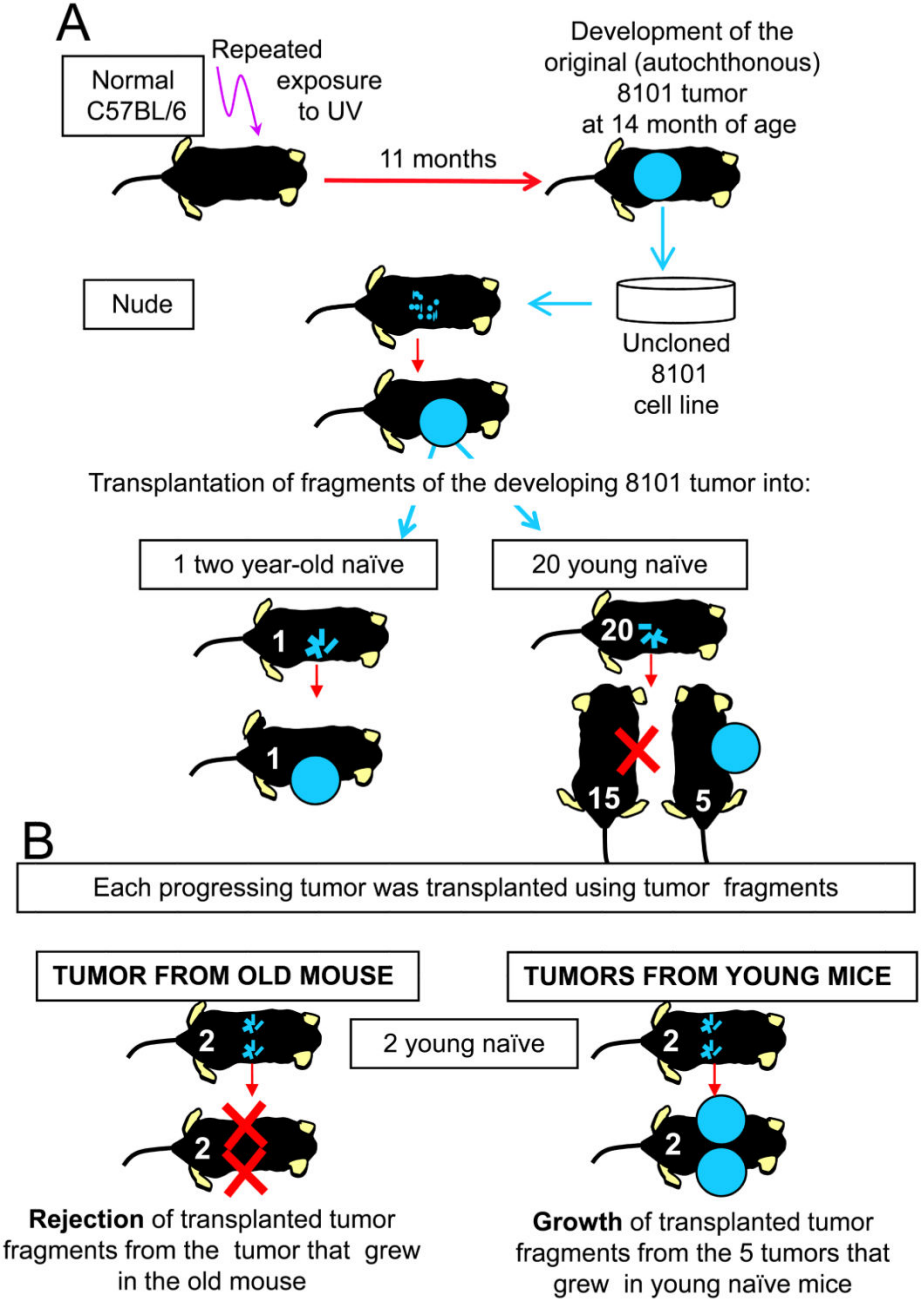
Cancer progressor variants are selected by young but not old naïve mice. **A.** Experimental design. Cryopreserved fragments of the autochthonous 8101 tumor were first adapted to culture and then injected into a nude C57BL/6 mouse that developed a tumor. Fragments of this tumor were transplanted into one old (2 year-old) and twenty young (2-3 month-old) normal euthymic C57BL/6 mice. Five of the twenty young and the old mouse failed to reject the tumor challenge. **B.** Analysis of the transplant behavior of each tumor found to be progressively growing in A by fragment transplantation into a new set of young naïve mice. Every tumor that had developed in a young naïve mouse grew again, whereas the tumor that grew in the old mouse was rejected.

Fragments of the cryopreserved autochthonous 8101 tumor were adapted to culture and then injected into an athymic nude C57BL/6 mouse (Figure 1A). Fragments of this tumor were transplanted into a total of 20 naïve 2-3 month-old euthymic C57BL/6 mice and 1 naïve 2 year-old normal euthymic C57BL/6 mouse that we had available in our colony. Most (15 of 20) young naïve mice rejected the inocula.^1^ The same results were obtained injecting cryopreserved fragments from the original 8101 tumor directly in naïve euthymic young animals (Suppl. Figure 1). Fragments of the 5 tumors that progressed in young mice were (i) adapted to culture for later analysis and (ii) transplanted into 2 month-old normal C57BL/6 mice, two bilateral injection sites per mouse. Fragments of the 8101 tumor grown in the athymic mouse grew in the 2 year-old mouse, and fragments of this tumor were also transplanted into two 2 month-old normal C57BL/6 mice bilaterally (Figure 1B). In contrast to the inocula of fragments from young mice, these inocula were rejected indicating the tumor that grew in the old host had not lost its antigenicity. We developed a mutation-specific PCR that identifies the single nucleotide substitution causing the immunodominant mutant p68 antigen (Suppl. Figure 2A). All progressors that grew in young mice retained the mutant gene, except for one that lost the mutant gene but kept the non-mutated p68 (PRO1A). However, analysis at mRNA level (Suppl. Figure 2B) showed that all the lines derived from young mice were negative for the transcript of the mp68 antigen. By contrast, the tumor that developed in the old mouse and was rejected when re-transplanted into young recipients (Figure 1) had retained expression of the mRNA of the mp68 antigen (Suppl. Figure 2B, right small panel). Tumors that lost expression of mp68 message were also resistant to lysis by 8101 specific T cells in a ^51^Cr-release assay (data not shown).

### The mp68 peptide binds to K^b^ with an extremely high affinity

To further understand why the mutant peptide had to be lost before 8101 could form tumors in young naïve mice, we analyzed the affinity of this peptide for the presenting MHC molecule K^b^. We found that the mutant peptide bound to K^b^ with an IC_50_ 0.48 nM (Table 1) and is therefore considered to be a very good antigen, comparable in affinity to viral peptides that protect 100% of mice from lethality when used for immunization against vaccinia virus infection (19).

### Lymphocytes from young immune but not from naïve or old immune mice reject very large tumors

**Figure 2:**
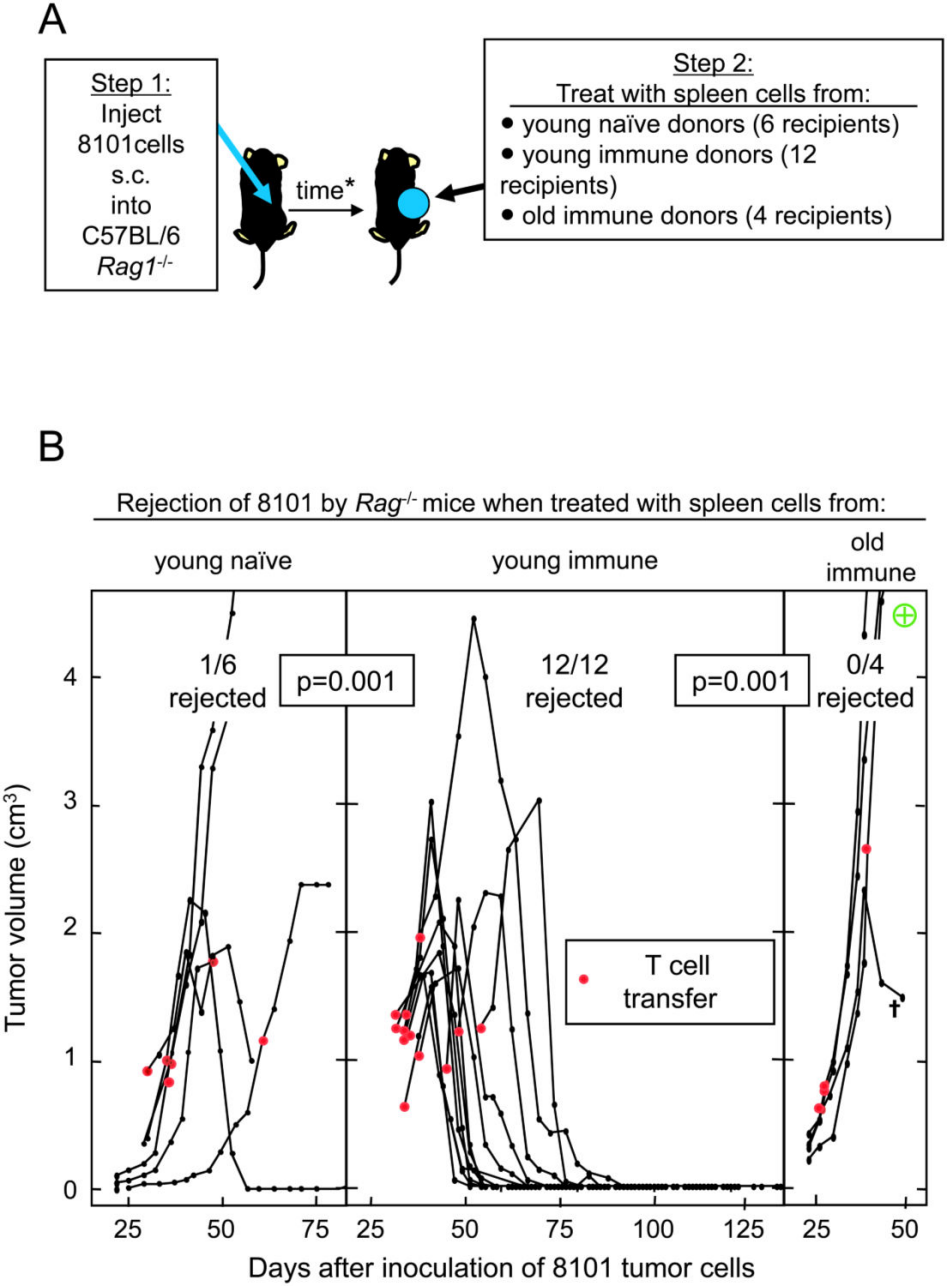
Adoptive transfer of spleen cells from young immune but not naïve or old immune mice leads to the eradication of large established 8101 tumors in *Rag1*^-/-^mice. **A.** Experimental design. **B.** Tumor-bearing *Rag1*^-/-^ C57BL/6 mice were treated by adoptive transfer of spleen cells (one spleen per recipient, around 1×10^8^ cells) from young naïve donors (3-4 month-old), young donors (3-4 month-old) immunized once with 2×10^7^ live 8101 cancer cells at the age of 2 months, or old immune mice (29 month-old) immunized when 4 month-old and boosted 2, 12 and 19 months later. Results are pooled from several experiments, one of which is sharing all three experimental groups and two sharing the young naïve and young immune groups. The ⊕ symbol in the right panel designates a tumor that was reisolated and found to express the mutant p68 gene by RT-PCR. * The average tumor size and duration of growth of 8101 (mean ± SD) at time of treatment was: 1117 ± 339 mm^3^ and 41 ± 11 d for the “naïve young” group; 1219 ± 315 mm^3^ and 39 ± 7 d for the “young immune”; 1203 ± 961 mm^3^ and 30 ± 6 d for the “old immune”.

We next designed experiments to determine how effectively spleen cells from naïve or immunized young or old mice could destroy clinical size 8101 tumors. The “uncloned” 8101 cell line (Figure 1) was grown in *Rag1*^-/-^ C57BL/6 mice (Figure 2A). Only 1 of the 6 8101 tumor-bearing *Rag1*^-/-^C57BL/6 mice rejected the established tumor when treated with naïve young C57BL/6 spleen cells (Figure 2B). Thus naïve spleen cells from young mice are usually ineffective in eradicating established 8101 tumors. By contrast, all 12 8101 tumor-bearing *Rag1*^-/-^ C57BL/6 mice rejected the established tumor when treated with spleen cells from young mice that had been immunized with live 8101 cancer cells. However, 4 mice that received spleen cells from older immunized donors (29 month-old), failed to reject the tumor. Remarkably, spleen cells from the older mice failed to eradicate the tumors even though 3 of 4 tumors treated with spleen cells from old immunized mice were significantly smaller than the average volume of tumors treated in the other two groups, and the age at which old and young donors were immunized for the first time was the same (details in Figure 2A and figure legend). The old donors had been last boosted 7 months before their spleen cells were used for the adoptive therapy. It is conceivable that the longer time interval between the last boost and use of the spleens for treatment was responsible for the failure of the spleen cells from the older immune mice to eradicate the tumors. In the subsequent experiments we controlled for these potential influences.

**Figure 3:**
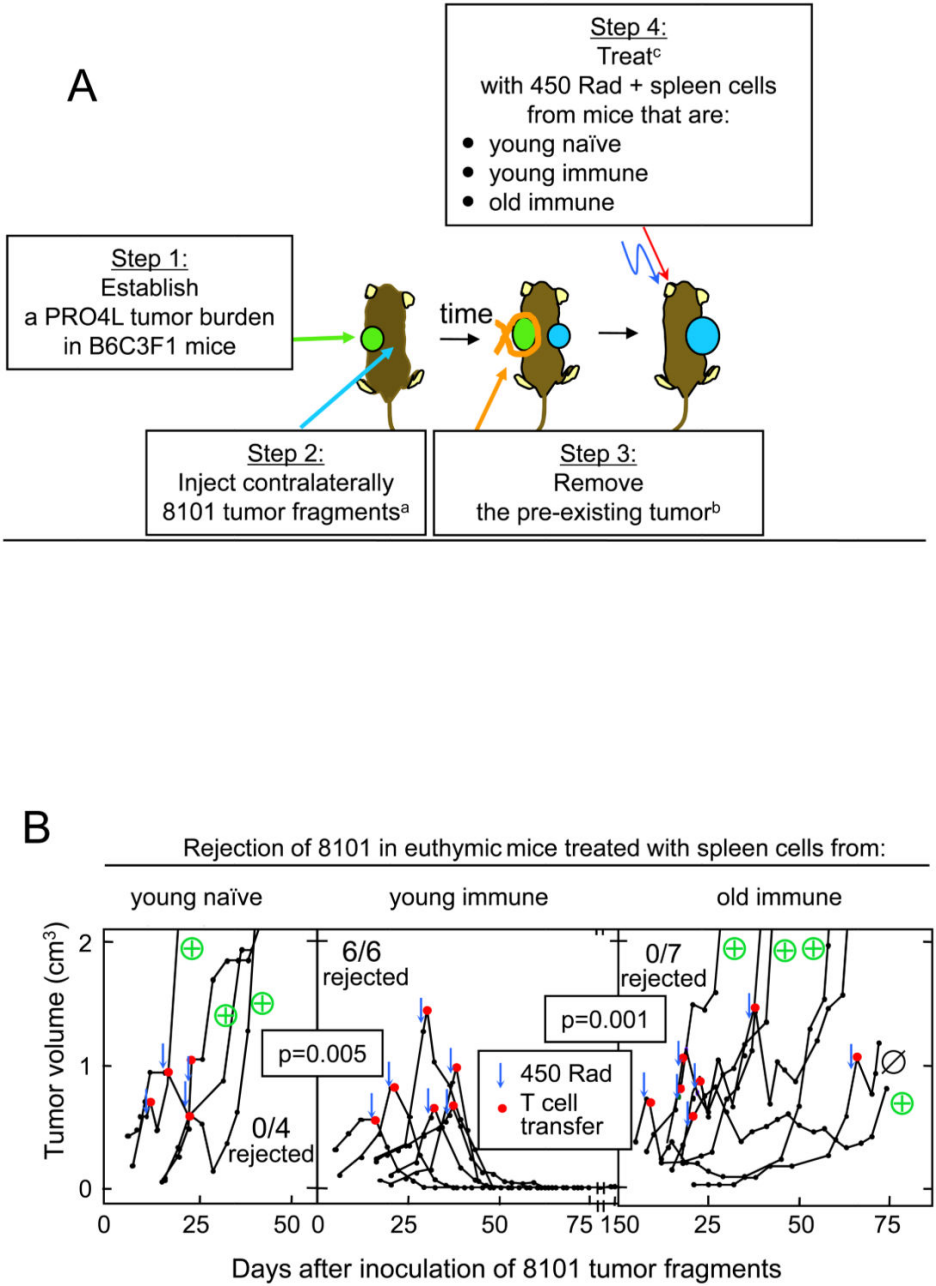
Adoptive transfer of spleen cells from young immune but not naïve or old immune mice leads to the eradication of large 8101 tumors grown in euthymic B6C3F1 mice. **A.** Experimental design. **B.** 8101-bearing euthymic B6C3F1 mice were treated by adoptive transfer of spleen cells (one spleen per recipient, around 1×10^8^ cells) from young naïve donors (3-4 month-old), young donors (4-8 month-old) immunized once with 2×10^7^ live 8101 cancer cells 2 months before transfer, or old immune mice (9-16 month-old) immunized when 2-3 month-old and boosted 2 months before transfer. Results are pooled from several experiments, one sharing all three experimental groups and two sharing the young naïve and young immune group. The ⊕ symbols in the right and left panels designate tumors that were reisolated and found to express the mutant p68 gene by RT-PCR; the antigen-loss variant is designated with Ø. ^a^ The pre-existent PRO4L tumor burden at time of 8101 inoculation was 539 ± 165 mm^3^ (mean ± SD) and had grown for an average of 21 ± 8 days. ^b^ The average size of PRO4L was 1577 ± 988 mm^3^ when strung at day 38 ± 10 of growth 8101 had grown for an average of 18 ± 10 days when the PRO4L tumor burden was removed. ^c^ The average tumor size and duration of growth of 8101 at time of treatment was: 738 ± 206 mm^3^ and 19 ± 6 d for the “naïve young” group; 845 ± 328 mm^3^ and 29 ± 9 d for the “young immune”; 909 ± 286 mm^3^ and 28 ± 19 d for the “old immune”.

We next determined how these findings obtained in T cell-deficient recipients applied when treating euthymic tumor-bearing mice. While 8101 tumor fragments are rejected in young euthymic B6C3F1 mice, tumor fragments failed to be rejected by these mice when they bore the unrelated C3H-derived tumor PRO4L on the contralateral side. 8101 grown under these conditions retains its rejection antigen (Suppl. Figure 3). B6C3F1 mice bearing only the 8101 tumor (after surgical removal of the PRO4L tumor) were treated with syngeneic spleen cells (Figure 3A). Once again, spleen cells from naïve young and old immunized mice failed to cause tumor rejection while all mice treated with young immune spleen cells rejected the tumors (Figure 3B), even though the time from last immunization/boost was equivalent for young and old immunized donors. All tumors treated with old immune cells grew out eventually and 4 of 5 tumors analyzed retained mp68 antigen expression (one grew as mp68-antigen loss variant).

### Old and young immunized mice have similar numbers of mp68-specific T cells after immunization with 8101 cancer cells

**Figure 4:**
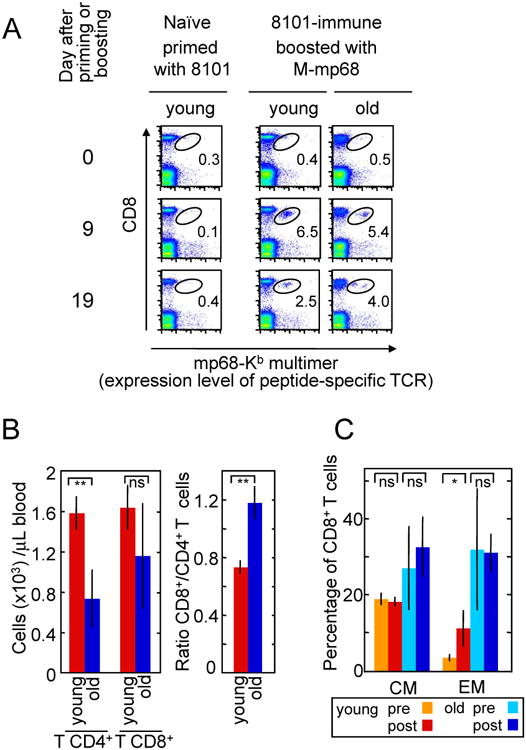
Old and young mice have similar numbers of mp68-specific CD8^+^ T cells but differ in number of CD4^+^ T cells and in the ability to increase the percentage of effector memory cells after boosting. **A.** Peripheral blood cells were isolated from naïve and immune young or old mice (5 mice per group) and the binding of mp68 peptide-loaded tetramers to CD8^+^ T cells was measured. The young (6 month-old) immune mice had been primed once at the age of 2 months whereas the old (16 month-old) immune mice had been primed at 2 months of age and boosted at 5 and 12 months of age. Day 0 of analysis corresponds to 4 months after immunization/last boosting respectively. The results are representative for 3 mice each, for young and old. M-mp68 is a cell line transfected to express very high levels of mp68 antigen. **B.** Absolute numbers of CD4^+^ and CD8^+^ T cells were determined in peripheral blood from old (14 month-old) and young (4 month-old) mice. An experiment representative of two is shown with data from 5 mice per group. **C.** The percentages of central memory (CM: CD62L^hi^/CD44^hi^), and effector memory (EM: CD62L^lo^/CD44^hi^) CD8^+^ T cells were determined in peripheral blood from old (16 month-old) and young (6 month-old) mice before (pre) and on day 9 after boosting (post) as in A. 4-5 mice per group were analyzed in total in two experiments pooled here. *p < 0.05; **p ≤ 0.01; ns, no significant.

We compared the frequency of mp68-specific CD8^+^ T cells in young versus old 8101-immune C57BL/6 mice (Figure 4A). Mice primed with 8101 cancer cells required a secondary challenge (boosting) with the mp68 antigen before an expansion of mutant p68-specific cells was detectable (day 9 and 19); a mp68-overexpressing MC57 cell line (M-mp68) was used to make the specific response more prominent. No differences in the frequency of mp68-specific T cells were detected in young compared to old mice. Thus, failure of old T cells to reject 8101 tumors cannot be explained by a lower frequency of mp68-specific CD8^+^ T cells.

### Young mice have more CD4^+^ T cells and respond to immunization with 8101 by increasing the percentage of effector memory cells

We then analyzed overall differences in T cell subpopulations between young and old mice. Old mice had lower absolute numbers of circulating T cells than young mice, and CD4^+^ T cells were the most affected subset. Thus, the ratio CD8^+^:CD4^+^ T cell was higher for old mice (Figure 4B). Furthermore, while percentage of regulatory T cells (T_reg_) among CD4^+^ T cells was increased in old mice, their absolute number was decreased (Suppl. Figure 4A). Old and young mice however differed in the composition of their CD8^+^ T cell pool. Consistent with what has been described before (20), young mice had more naïve T cells, while the percentage of memory cells in the old mice was higher (Suppl. Figure 4B). Interestingly, after boosting with the mp68 antigen-expressing cancer cells, the percentage of effector memory CD8^+^ T cells increased significantly in young mice (Figure 4C). In contrast, the percentage of effector memory cells remained unaltered in old mice, and a tendency to increase was observed in the percentage of central memory cells (although not statistically significant).

## Discussion

Probably all cancers have mutant genes and express epitopes that are not self. Such epitopes may bind to MHC molecules with high affinity, stimulate immunity effectively and also serve as targets for effector cells. Some tumor-specific somatic mutations affect genes expressed on the surface of cancer cells and are recognized by tumor-specific antibodies (21-23). The fact that most tumor-specific somatic mutations however seem to affect genes not expressed on the surface membrane of cancer cells (24-27) should not matter, for such antigens could be effectively presented as mutant peptide/MHC molecule complexes on the surface of cancer cells or after cross-presentation on the surface of stromal cells in the tumor (3, 28, 29).

As we show for the immunodominant tumor-specific antigen of the 8101 cancer, mutant epitopes such as the mutant p68 peptide may bind to MHC Class I molecules with very high affinity (below 1 nM). Unlike transfected and overexpressed model target antigens, this antigen originated during tumorigenesis in the autochthonous 8101 cancer. We further show in our study that this antigen was always lost before the cancer could grow in immunocompetent young mice. In contrast, 8101 cancers expressed the antigen in the old mouse in which it originated and in the old mouse receiving tumor fragments. Antigen-negative variants were probably present in tumors growing in both young and old hosts; however, only in young mice immunological pressure selected for mp68-negative variants, whereas in the old mice the variants remained a minority. Since the majority of common cancers are first diagnosed in older individuals, most human cancers may have retained strong antigens such as mp68. Thus, our results are consistent with the possibility that old age should favor retention of strong rejection antigens.

Two conditions needed to be fulfilled by the donor of immune cells for successful therapy of 8101: to be young and to be immunized against the tumor being treated (truly individualized immunization and therapy). How could these conditions be fulfilled in patients?

Adoptively transferred cells should come from young donors. The age of the individual at the moment of immunization is known to be critical for effective vaccination (30). However, we did not find preclinical studies comparing the efficacy of lymphocytes from old and young donors in adoptive transfer. Our studies show that even when the first immunization took place when donors were young, immune lymphocytes from aged mice lost their efficacy for treatment of tumors upon adoptive transfer into young hosts. Interestingly, our cut-off point for age of donors was 9 months, which corresponds to a middle-age mouse. Most cancer patients eligible for adoptive T cell therapy might also be between 40-60 (13-17). Importantly, T cells must be effective in the “old” environment of the patient (31). Our experiments tested the efficacy of transferred T cells only in young tumor-bearing hosts, and future experiments need to test whether adoptively transferred young immune spleen cells can be effective in old tumor-bearing mice. Previous studies (31) indicated that adoptively transferred young T cells proliferate poorly in the environment of old hosts. This problem could possibly be overcome by treating the old host with anti-type I interferon antibodies as suggested by Sprent and co-workers (31).

We immunized tumor-free syngeneic mice for adoptive transfer. Finding tumor-free human donors who are syngeneic will be impossible (unless an identical twin was available). Haploidentical patient-related tumor-free donors are more readily available and younger if they are children. It needs to be explored how lethal graft-versus-host effects by T cells from such donors can be circumvented. However, the success of strategies as allogeneic Epstein-Barr virus (EBV) nuclear antigen (EBNA)-specific T cells sharing major MHC allele with the patient, that can treat successfully post-transplant lymphoproliferative disease (32) and even bulky EBV-positive lymphomas (33, 34), sets grounds for hope.

Why do old splenocytes fail? When we compared T cell compartments in old and young mice, we found two main differences: old mice (i) had less T cells (especially CD4^+^ T cells) and (ii) did not increase the percentage of effector memory CD8^+^ T cells after boosting with the mp68 antigen, in contrast to young mice. This is consistent with the reduction in turnover observed in memory CD8^+^ T cells from aged mice (31). Proliferation and infiltration of effector cells must happen for tumor rejection. CD4^+^ T cells have been shown to be essential for expansion of memory cells (35) for tumor infiltration by CD8^+^ T cells (36) and optimal function of CD8^+^ T cells at the effector phase (36, 37). Aged CD4^+^ T cells form defective immunological synapses (38). Thus, a defective response of the memory CD8^+^ T cells and ineffective help by CD4^+^ T cells could explain why old immune splenocytes failed to reject 8101 tumors.

Young naïve euthymic splenocytes protect B6C3F1 mice against a 8101 tumor challenge. However, once the tumor is well established, transfer of naïve spleen cells is no longer effective. Studies of the immune response to sporadic cancers expressing SV40 T antigen suggest that once the cancer is established it is no longer immunizing but tolerizing (39). These cancers lack the pro-inflammatory type I cytokine environment caused by the initial injury of fragment or cancer cell inoculation. Also, autochthonous newly arising or long-established transplanted cancers probably have quite different stromal composition of bone marrow-derived cells, fibroblastic cells and extracellular matrix (for review see (40)). In addition, tumor-induced regulatory T cells and myeloid-derived suppressor cells have been demonstrated to suppress naïve T cell responses (41-44).

Together, our studies show that clinical size solid tumors can be eradicated by adoptive transfer of spleen cells from young immunized donors without requiring artificially transfected antigens or TCR-transgenic T cells. Our model avoided the use of serially transplanted tumors; by contrast, we used cryopreserved original tumor fragments and a primary cell line. Also, the cancers we treated were truly long-established in the host. A systematic analysis of recent studies confirms the century old assertion (45) that many procedures are effective early after cancer cell inoculations but not later (2). Adoptive T cell transfer was singled out as the most effective approach at later stages, consistent with findings of clinical studies. But even for adoptive T cell therapy, we show here, stringent requirements must be fulfilled to eradicate clinically relevant tumors.

## Materials and Methods

### Mice, cell lines, and reagents

C57BL/6 and C57BL/6 *Rag1^-/-^* mice were purchased from The Jackson Laboratory. B6C3F1 mice were obtained from Charles River Laboratories. C3H *Rag2^-/-^* mice were obtained from Douglas Hanahan (University of California, San Francisco, California). All mice were maintained in a specific pathogen-free barrier facility at the University of Chicago according to the Institutional Animal Care and Use Committee guidelines.

PRO4L was originated in a C3H/HeN mouse and has been previously described (46). 8101 originated in UV-treated C57BL/6 and has been described (18, 27). P. Ohashi (University of Toronto, Toronto, Ontario, Canada), with permission of H. Hengartner (University Hospital Zurich, Zurich, Switzerland), provided the MC57G methylcholanthrene-induced, C57BL/6-derived fibrosarcoma (MC57). MC57-mp68-EGFP (M-mp68) was generated by retroviral transduction. Phoenix-ampho cells (47) were transfected with pMFG-(mp68-AAY)_3_-EGFP using the CalPhos Mammalian Transfection Kit (Clontech, Mountain View, CA). Repeated rounds of transduction of MC57 with viral supernatants and FACS-sorting derived the highly peptide/fluorescent protein-expressing line.

pMFG-(mp68-AAY)_3_-EGFP was constructed by inserting annealed oligonucleotides (IDT, Coralville, IA) encoding triple SNFVFAGI-AAY repeats into the NcoI-linearized (NEB, Ipswich, MA) pMFG-EGFP vector (kindly provided by R.C. Mulligan (Children's Hospital Boston, Boston, MA, (48)).

### Tumor challenge and treatment

For the experiments in *Rag1*^-/-^ mice, 10^7^ 8101 cells were injected subcutaneously (s.c.) onto the shaved back of mice. Tumor volumes were measured along three orthogonal axes (a, b, and c) every 3 to 4 days and tumor volume was calculated as abc/2. Mice were treated intraperitoneally with naïve or immune splenocytes (one spleen per recipient, around 1 ×10^8^ cells). For the experiments in euthymic B6C3F1 mice, PRO4L tumors were grown in C3H *Rag2*^-/-^ mice and were implanted s.c. as viable 1 mm^3^ fragments with a 12- gauge trocar (1 full trocar load) into the left flank of anesthetized B6C3F1 mice. Once PRO4L was established, 8101 tumors grown in C57BL/6 *Rag1*^-/-^ mice were implanted in the right flank as fragments. Once 8101 was established (for details see Figure 3A), PRO4L tumor was removed by tying off the tumor at its base (“stringing”).

For the generation of memory T cells, 2 × 10^7^ 8101 cancer cells were injected s.c. into the flanks of B6C3F1 mice or C57BL/6 and their spleens were used for adoptive transfer.

### PCR analysis for mutant p68 expression

Genomic DNA and total RNA were isolated from cancer cell lines using QIAamp DNA mini and RNeasy mini kits. RNA was treated with DNase I (Roche) and reverse transcriptase (New England Biolabs, Beverly, MA) to synthesize the cDNA. PCR was performed on the genomic DNA or cDNA using the following primers: Forward 5-GGGGATCCGCCATGAAGGACGATCGTCGTGACAG-3 and reverse primer 5 -AGAATACCCTGTTGGCATGG-3 amplify a 425 bp fragment of the murine p68 RNA helicase. Forward primer 5 -GGAGCTTTGGAAGTAATTTTTGTTTT-3 was designed to detect specifically a point mutation at the nucleotide position 1812 of p68, and amplifies a 290 bp fragment only if the mutation is present. Vectors containing mutant and wild type p68 minigenes on the pIRES-EGFP vector backbone (Clontech, Mountain View, CA) were used as controls.

### T cell analysis in peripheral blood

Percentages of T cell subpopulations were measured in peripheral blood after lysis of red blood cells. For the determination of absolute numbers of cells, AccuCount Rainbow beads (Spherotech, Lake Forest, IL) were used according to the manufacturer's instructions. For the analysis of the frequency of mp68-specific T cells, old or young immune or naïve mice received 7 – 10 × 10^6^ 8101 or MC57-mp68-EGFP cancer cells and were subsequently bled at days 5, 9 and 19. Analysis before cancer cell injection served to determine the background staining (day 0).

### Flow cytometry

Cells were stained using anti-CD3, CD4, CD8, CD44 and anti-CD62L mAb (all from BioLegend or eBioscience). Specific T cells were detected with a mp68-K^b^ tetramer (NIH Tetramer Core Facility). T_reg_ were analyzed using the mouse regulatory T cell staining kit from eBioscience. Flow cytometry data were acquired on FACSCalibur or FACSCanto machines (BD) and data were analyzed using FlowJo (Tree Star, Ashland, OR) software. Cell sorting was performed using FACSAria (BD) or MoFlo-HTS (Beckman Coulter, Brea, CA) at the Flow Cytometry Facility of The University of Chicago.

### MHC peptide binding assays

MHC purification, and quantitative assays to measure the binding affinity of peptides to purified H2-K^b^, H2-D^b^, and HLA-A*0201 molecules were performed as previously described (49, 50).

### Statistical analysis

Results of treatment of small groups of mice were analyzed using the two-tailed p-value calculated by Fisher's exact test using Stata. (p ≤ 0.05 is considered significant, p ≤ 0.01 highly significant). Differences between two sets of data were analyzed using the Student's t-test (paired for CD8^+^ T cell populations in the same mouse; unpaired for numbers and percentages of CD4^+^ and CD8^+^ T cells and T_reg_ in different groups of mice).

## Supplementary Material

**Supplementary Figure 1**. A cancer progressor variant is selected by a young mouse upon injection of cryopreserved 8101 original tumor fragments. **A.** Experimental design. Cryopreserved fragments of the autochthonous 8101 tumor were injected into a nude C57BL/6 mouse that developed a tumor. Fragments of this tumor were transplanted into ten young (2-3 month-old) normal euthymic C57BL/6 mice. One of the ten young mice failed to reject the tumor challenge. **B.** The tumor that had developed in a young naïve mouse grew upon transplantation into new naïve C57BL/6 mice.
**Supplementary Figure 2.** Loss of expression of the mutant p68 (mp68) rejection antigen by all 8101 variants that grew progressively in naïve young mice but retention by 8101 tumor that developed in the old recipient. **A.** mp68-specific PCR analysis on genomic DNA **B**. mp68-specific RT-PCR. All variants except PRO1A retained the mutant gene but lost the mRNA indicating the variants had heritably shut off the transcription of the mutant gene. As an internal control, a fragment of p68 was amplified on each sample using primers not specific for the mutation (p68). 4102 is an unrelated cell line used as a specificity control. Variants PRO1 and PRO1A developed in the same young mouse (18).
**Supplementary Figure 3**. 8101 tumors grown in B6C3F1 euthymic mice bearing a preexistent PRO4L tumor are mp68 antigen-positive. 8101 tumors developed in B6C3F1 mice that had a pre-existent PRO4L tumor at the time of injection of 8101 tumor fragments. After stringing of the PRO4L tumor, the 8101 tumor continued to grow. Re-transplantation of the 8101 tumors into 4 naïve young B6C3F1mice bilaterally led to rejection of all 8 inocula.
**Supplementary Figure 4**. A. Percentages and absolute numbers of regulatory CD4^+^ T cells (T_reg_) in the peripheral blood of young (5 month-old) and old (15 month-old) mice. The % of T_reg_ was measured as %CD25^+^FoxP3^+^ in the gated CD4^+^ T cell population (CD3^+^CD4^+^). The plots show data from 1 experiment with 4 mice per group. **B.** The percentages of naïve (CD62L^hi^/CD44^lo^), and memory CD8^+^ T cells [central memory (CM: CD62L^hi^/CD44^hi^), and effector memory (EM: CD62L^lo^/CD44^hi^)] were determined in peripheral blood from old (16 month-old) and young (6 month-old) mice. Central memory and effector memory cells were considered together as the “memory” population. 4-5 mice per group were analyzed in two experiments pooled here. *p < 0.05; **p ≤ 0.01; ns, no significant.

## Figures and Tables

**Table 1:** The mutant p68 peptide binds MHC class I K^b^ with an affinity comparable to that of viral peptides that, when used for immunization, protect 100 % of mice against a lethal challenge with vaccinia.

Designation	Sequence	MHC	Affinity of peptide for MHC (IC_50_ [nM])^a^	Geometric standard deviation (times/divide)
mp68 (547-554, 5F)	SNFVFAGI	K^b^	0.48	2.63
p68 (547-554)	SNFVSAGI	K^b^	22.00	2.06
A23R (297-305)^b^	IGMFNLTFI	D^b^	0.34^c^	2.82
A6L (265-272)^b^	YTLIYRQL	K^b^	6.00^c^	4.43

a IC_50_ values are the geometric mean of 5 or more experiments.

b Vaccinia virus strain Copenhagen protein nomenclature

c Published in (19).

## Translational Relevance

Preclinical models of immunotherapy are often successful at early stages of cancer, but fail when tumors become closer to the level of development at which they would be discovered in a clinical setting. Adoptive T cell therapy stands out as the most successful immunotherapy for cancer. However, the characteristics that determine T cell efficacy are insufficiently understood. In a genetically non-manipulated preclinical model of long-established tumors, we found that T cells must be immune against the tumor antigens, and, importantly, come from young individuals. These findings show for the first time that the age of the donor is critical for the efficacy of adoptively transferred cells. Since most tumors develop in old age, this implies that for adoptive T cell therapy of solid tumors, optimal efficacy might be achieved by T cells harvested from patient-related healthy young donors immunized against the tumor antigens, i.e., by individualized immunization and therapy.

## Footnotes

1 We previously reported this group of 20 young naïve mice challenged with 8101 tumor fragments with 15 rejecting and 5 growing the inoculum progressively. However, only 1 of these 5 progressing tumors had been analyzed previously to determine if progressive growth was heritably acquired (18). The remaining 4 progressing tumors are first analyzed here. Also, none of these 5 variants had previously been analyzed by PCR for the mechanism of antigen loss (i.e. at DNA or RNA level).
