# Supplementary material for: Spleen cells from young but not old immunized mice eradicate large established cancers
Source: Clin Cancer Res. Author manuscript; Available in PMC 2017 Mar 16. (PMC5354938; doi:10.1158/1078-0432.CCR-12-0127)
Supplement: Supplementary file 1 — Supplementary Figure 1. A cancer progressor variant is selected by a young mouse upon injection of cryopreserved 8101 original tumor fragments. A. Experimental design. Cryopreserved fragments of the autochthonous 8101 tumor were injected into a nude C57BL/6 mouse that developed a tumor. Fragments of this tumor were transplanted into ten young (2-3 month-old) normal euthymic C57BL/6 mice. One of the ten young mice failed to reject the tumor challenge. B. The tumor that had developed in a young naïve mouse grew upon transplantation into new naïve C57BL/6 mice. Supplementary Figure 2. Loss of expression of the mutant p68 (mp68) rejection antigen by all 8101 variants that grew progressively in naïve young mice but retention by 8101 tumor that developed in the old recipient. A. mp68-specific PCR analysis on genomic DNA B. mp68-specific RT-PCR. All variants except PRO1A retained the mutant gene but lost the mRNA indicating the variants had heritably shut off the transcription of the mutant gene. As an internal control, a fragment of p68 was amplified on each sample using primers not specific for the mutation (p68). 4102 is an unrelated cell line used as a specificity control. Variants PRO1 and PRO1A developed in the same young mouse (18). Supplementary Figure 3. 8101 tumors grown in B6C3F1 euthymic mice bearing a preexistent PRO4L tumor are mp68 antigen-positive. 8101 tumors developed in B6C3F1 mice that had a pre-existent PRO4L tumor at the time of injection of 8101 tumor fragments. After stringing of the PRO4L tumor, the 8101 tumor continued to grow. Re-transplantation of the 8101 tumors into 4 naïve young B6C3F1mice bilaterally led to rejection of all 8 inocula. Supplementary Figure 4. A. Percentages and absolute numbers of regulatory CD4+ T cells (Treg) in the peripheral blood of young (5 month-old) and old (15 month-old) mice. The % of Treg was measured as %CD25+FoxP3+ in the gated CD4+ T cell population (CD3+CD4+). The plots show data from 1 experiment with 4 mice p [file NIHMS850815-supplement.pdf]

SUPPLEMENTARY FIGURE LEGENDS

Supplementary Figure 1. A cancer progressor variant is selected by a young mouse upon injection of cryopreserved 8101 original tumor fragments. **A.** Experimental design. Cryopreserved fragments of the autochthonous 8101 tumor were injected into a nude C57BL/6 mouse that developed a tumor. Fragments of this tumor were transplanted into ten young (2-3 month-old) normal euthymic C57BL/6 mice. One of the ten young mice failed to reject the tumor challenge. **B.** The tumor that had developed in a young naïve mouse grew upon transplantation into new naïve C57BL/6 mice.

Supplementary Figure 2. Loss of expression of the mutant p68 (mp68) rejection antigen by all 8101 variants that grew progressively in naïve young mice but retention by 8101 tumor that developed in the old recipient. **A.** mp68-specific PCR analysis on genomic DNA **B**. mp68-specific RT-PCR. All variants except PRO1A retained the mutant gene but lost the mRNA indicating the variants had heritably shut off the transcription of the mutant gene. As an internal control, a fragment of p68 was amplified on each sample using primers not specific for the mutation (p68). 4102 is an unrelated cell line used as a specificity control. Variants PRO1 and PRO1A developed in the same young mouse (18).

Supplementary Figure 3. 8101 tumors grown in B6C3F1 euthymic mice bearing a preexistent PRO4L tumor are mp68 antigen-positive. 8101 tumors developed in B6C3F1 mice that had a pre-existent PRO4L tumor at the time of injection of 8101 tumor fragments. After stringing of the PRO4L tumor, the 8101 tumor continued to grow. Re-transplantation of the 8101 tumors into 4 naïve young B6C3F1mice bilaterally led to rejection of all 8 inocula.

Supplementary Figure 4. A. Percentages and absolute numbers of regulatory $CD4^+$ T cells (T_{reg}) in the peripheral blood of young (5 month-old) and old (15 month-old) mice. The % of T_{reg} was measured as $\%CD25^+FoxP3^+$ in the gated $CD4^+$ T cell population ($CD3^+CD4^+$). The plots show data from 1 experiment with 4 mice per group. **B.** The percentages of naïve ($CD62L^{hi}/CD44^{lo}$), and memory $CD8^+$ T cells [central memory (CM: $CD62L^{hi}/CD44^{hi}$), and effector memory (EM: $CD62L^{lo}/CD44^{hi}$)] were determined in peripheral blood from old (16 month-old) and young (6 month-old) mice. Central memory and effector memory cells were considered together as the "memory" population. 4-5 mice per group were analyzed in two experiments pooled here. * $p < 0.05$; ** $p \leq 0.01$; ns, no significant.

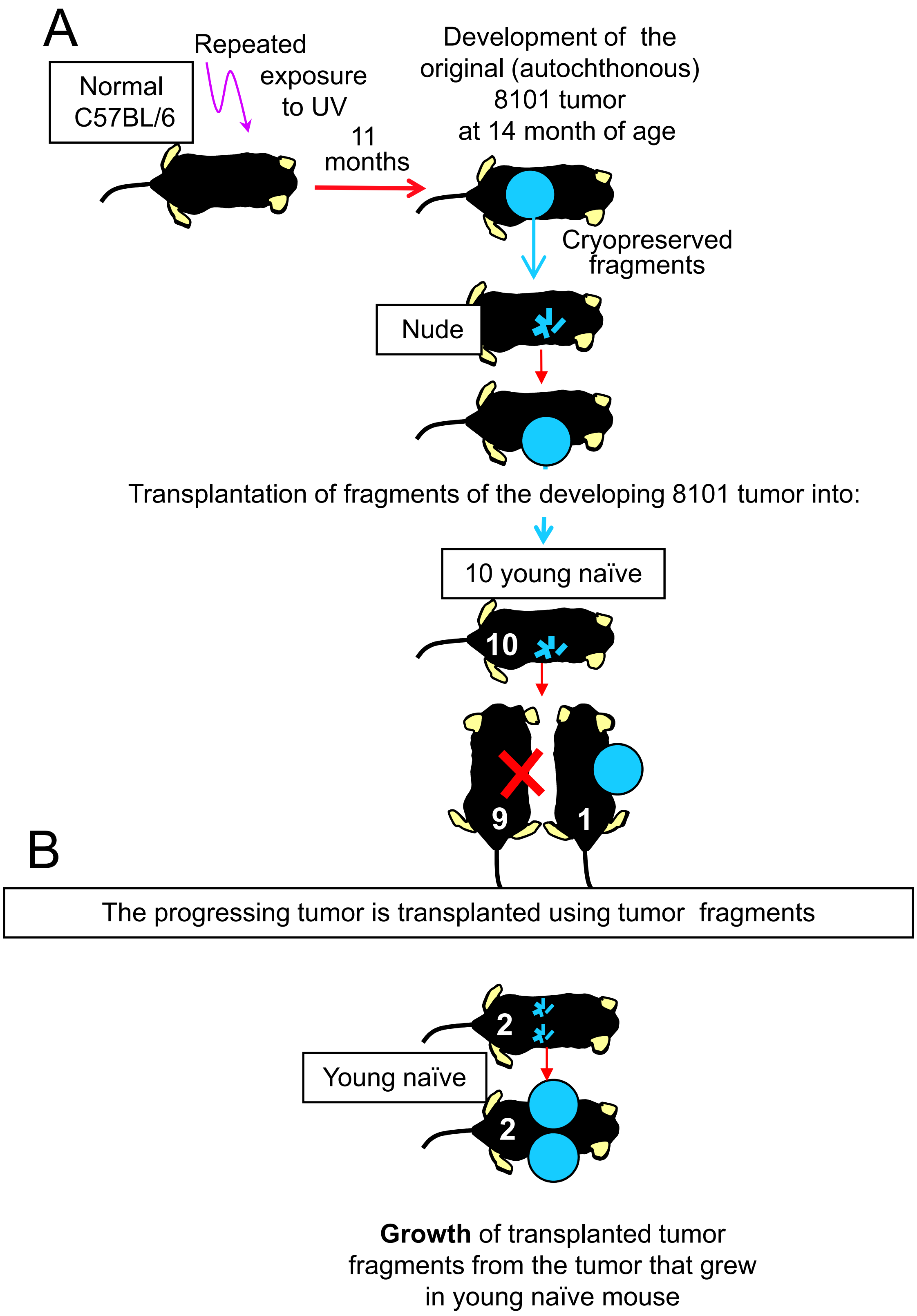

Schreiber *et al.* Supplementary Figure 1

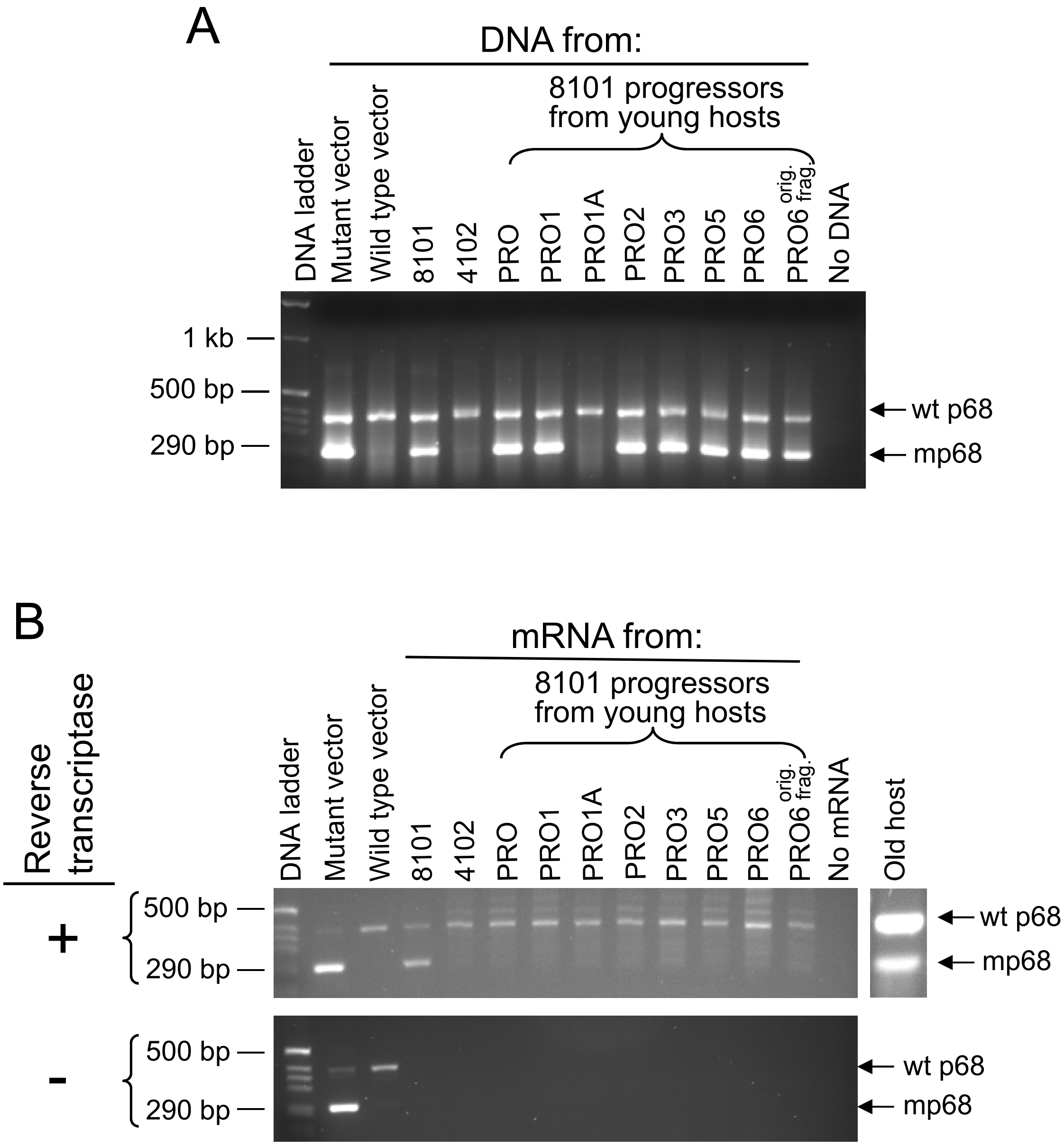

Schreiber *et al.* Supplementary Figure 2

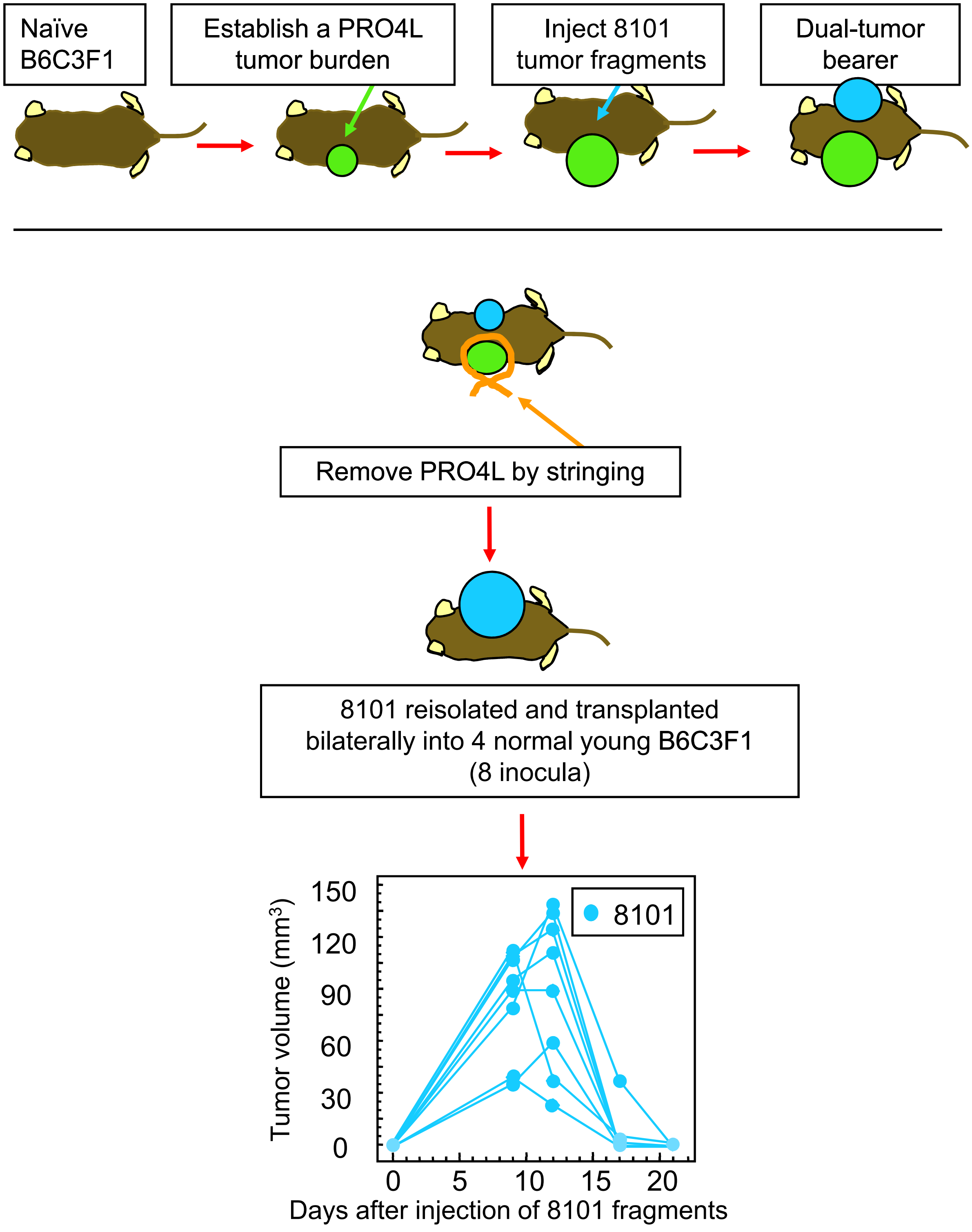

<u>Conclusion</u>:
All eight 8101 inocula retained the rejection antigen as evidenced by their rejection by naïve young B6C3F1 mice.

Schreiber et al. Supplementary Figure 3

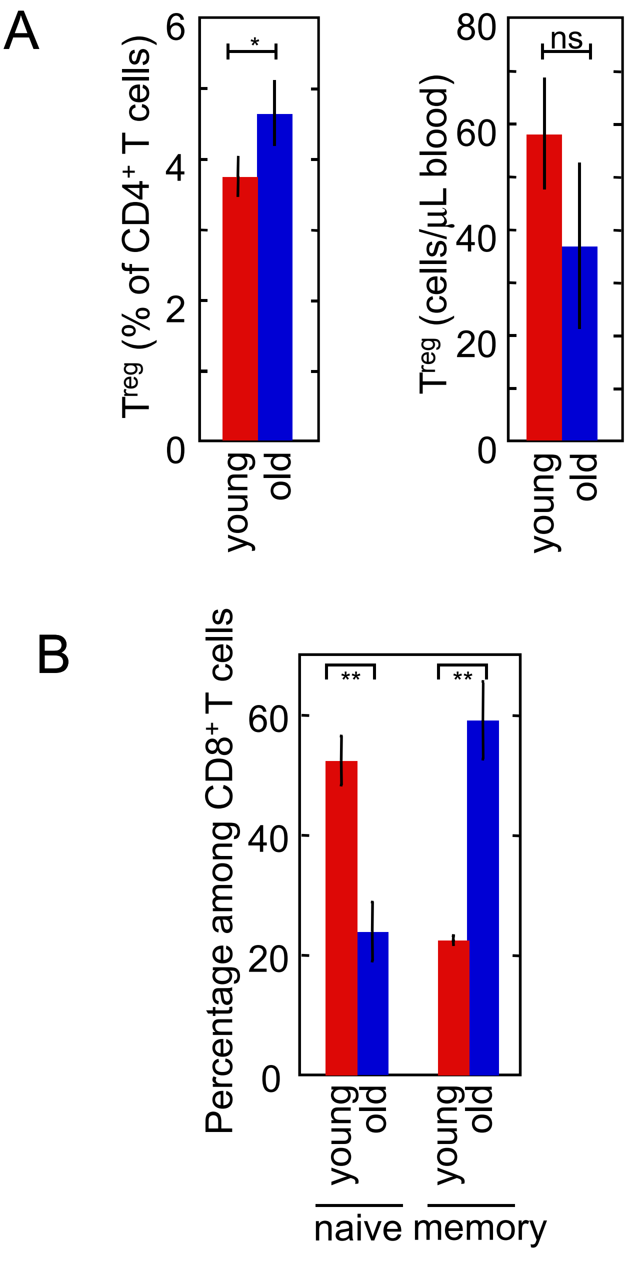

Schreiber et al. Supplementary Figure 4
